# Interferon-Linked Lipid and Bile Acid Imbalance Uncovered in Ankylosing Spondylitis in a Sibling-Controlled Multi-Omics Study

**DOI:** 10.3390/ijms26167919

**Published:** 2025-08-16

**Authors:** Ze Wang, Yi Huang, Ziyu Guo, Jianhua Sun, Guoquan Zheng

**Affiliations:** 1 Shihezi University School of Medicine, Shihezi University, Shihezi 832003, China; 20222014103@stu.shzu.edu.cn; 2 Nankai University School of Medicine, Nankai University, Tianjin 300071, China; huangyi@mail.nankai.edu.cn; 3 Laboratory of Medical Molecular Virology (MOE/NHC/CAMS), Shanghai Medical College, Fudan University, Shanghai 200433, China; 24111010053@m.fudan.edu.cn

**Keywords:** ankylosing spondylitis, dual-omics, RNA-seq, metabolomics

## Abstract

Ankylosing spondylitis (AS) displays wide inter-patient variability that is not accounted for by HLA-B27 alone, suggesting that additional immune and metabolic modifiers contribute to disease severity. Using a genetically matched design, we profiled peripheral blood mononuclear cells from two brother pairs discordant for AS severity and one healthy brother pair. Strand-specific RNA-seq was analyzed with a family-blocked DESeq2 model, while untargeted metabolites were quantified using gas chromatography–mass spectrometry (GC-MS) and liquid chromatography–mass spectrometry (LC-MS). Differential features were defined as follows: differentially expressed genes (DEGs) (|log_2_FC| ≥ 1 and FDR < 0.05) and metabolites (VIP > 1, FC ≥ 1.2, and BH-adjusted *p* < 0.05). Pathway enrichment was performed with KEGG and Gene Ontology (GO). A total of 325 genes were differentially expressed. Type I interferon and neutrophil granule transcripts (e.g., *IFI44L*, *ISG15*, *S100A8/A9*) were markedly up-regulated, whereas mitochondrial β-oxidation genes (*ACADM*, *CPT1A*, *ACOT12*) were repressed. Metabolomics revealed 110 discriminant features, including 25 MS/MS-annotated metabolites. Primary bile acid intermediates were depleted, whereas oxidized fatty acid derivatives such as 12-Z-octadecadienal and palmitic amide accumulated. Spearman correlation identified two antagonistic modules (i) interferon/neutrophil genes linked to pro-oxidative lipids and (ii) lipid catabolism genes linked to bile acid species that persisted when severe and mild siblings were compared directly. Enrichment mapping associated these modules with viral defense, neutrophil degranulation, fatty acid β-oxidation, and bile acid biosynthesis pathways. This sibling-paired peripheral blood mononuclear cell (PBMC) dual-omics study delineates an interferon-driven lipid–bile acid axis that tracks AS severity, supporting composite PBMC-based biomarkers for future prospective validation and highlighting mitochondrial lipid clearance and bile acid homeostasis as potential therapeutic targets.

## 1. Introduction

Ankylosing spondylitis (AS) is a seronegative spondylarthritis that preferentially affects young men and ultimately leads to spinal ankylosis, impaired mobility, and a marked reduction in quality of life [1]. Epidemiological surveys place its prevalence between 0.1% and 1.4% worldwide, with a substantial economic burden arising from work-productivity loss and the eventual need for spinal-corrective surgery [2]. Although carriage of the class I allele *HLA-B27* represents the single strongest genetic risk factor, it explains less than half of the estimated heritability, and its penetrance is highly variable across ethnicities [3]. Genome-wide association studies have extended the risk landscape to dozens of non-HLA loci—chiefly genes involved in antigen presentation, innate immunity and amino-acid metabolism—but how these variants translate into the complex serological and radiographic phenotypes of AS remains incompletely understood [4,5,6].

At the molecular level, circulating cytokine profiles indicate a chronic, mixed innate-adaptive inflammatory state typified by *TNF-α*, *IL-17*/*IL-23*, and type I interferon activity [7]. Parallel metabolomic surveys have reported altered lipid β-oxidation, tryptophan-kynurenine catabolism, and bile acid turnover in the serum or faces of AS patients [8,9,10]. However, most studies have interrogated either the transcriptome or the metabolome in isolation, precluding the discovery of cross-layer interactions that may pinpoint mechanistic disease hubs. Integrated multi-omics analyses, combining gene expression data with global metabolite readouts, have successfully exposed pathogenic circuits in rheumatoid arthritis, systemic lupus erythematosus, and inflammatory bowel disease [11,12,13], but remain scarce in spondylarthritis. To our knowledge, no study has yet combined untargeted PBMC-based RNA-seq with metabolomics in AS.

Such integrative approaches are clinically attractive for three reasons. First, PBMCs can be obtained minimally invasively and repeatedly, facilitating longitudinal monitoring. Second, joint modelling of transcripts and metabolites can reveal regulatory relationships (e.g., enzyme–substrate pairs, cytokine–lipid axes) that single-layer analyses miss. Third, composite multi-omics signatures often achieve superior diagnostic or prognostic performance compared with individual biomarkers, an important consideration as treat-to-target strategies gain traction in AS management [12].

Here, we conducted an exploratory, sibling-paired, PBMC-based dual-omics study—combining strand-specific RNA-seq with untargeted GC-MS/LC-MS—to (i) delineate transcriptomic and metabolomic alterations that distinguish AS individuals from healthy siblings; (ii) identify cross-layer gene–metabolite correlations that converge on immunometabolic pathways; and (iii) prioritize lipid- and bile acid-linked circuits for future validation. Leveraging brother pairs minimizes inter-individual genetic noise and enhances sensitivity to disease-related signals, while all findings are interpreted as hypothesis generating given the small, male-only cohort.

## 2. Results

### 2.1. Global Quality Assessment and Sample Clustering

**Figure 1:**
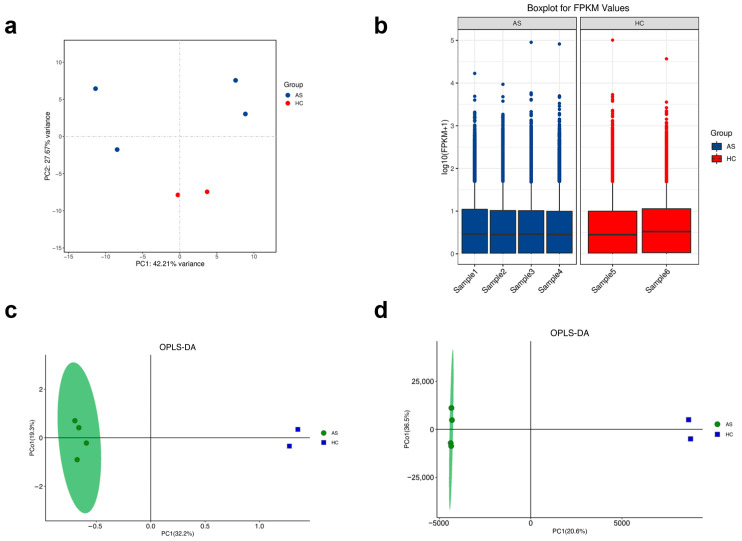
Global transcriptomic and metabolomic discrimination between AS and HC. (**a**) PCA of variance-stabilized RNA-seq counts; AS (blue) and HC (red) separate along PC1 (42.1%). (**b**) Box-and-whisker plots of log10-FPKM values show comparable library depth. (**c**) GC-MS OPLS-DA score plot (R^2^Y = 0.93; Q^2^ = 0.71) with 95% confidence ellipse. (**d**) LC-MS OPLS-DA score plot (R^2^Y = 0.95; Q^2^ = 0.76).

Variance-stabilized PCA showed that biological status is the dominant driver of transcriptomic variability. The four AS libraries grouped on the negative side of PC1, whereas the two HCs grouped on the positive side. PC1 and PC2 explained 42.1% and 27.7% of total variance, respectively (Figure 1a). Log10-FPKM distributions were nearly super-imposable across all six libraries (Figure 1b), confirming uniform sequencing depth and negligible batch effects.

Untargeted metabolomics produced concordant patterns. GC-MS OPLS-DA yielded a robust model (R^2^Y = 0.93; Q^2^ = 0.71) in which AS and HC specimens formed non-overlapping clusters along the first predictive component, enclosed within the 95% confidence ellipse (Figure 1c). The corresponding S-plot revealed that a focused set of ions with high covariance and correlation drives the separation, nominating them as candidate markers (Figure S1).

Independent LC-MS profiling reinforced these findings. Its OPLS-DA model achieved R^2^Y = 0.95 and Q^2^ = 0.76, again cleanly partitioning the two groups (Figure 1d). The LC-MS S-plot highlighted high-VIP metabolites that mirror those emerging from the GC-MS analysis (Figure S2). Instrument performance was stable: pooled QC injections across 9655 LC features displayed a median RSD of 12.7%, well below the 15% threshold.

Collectively, these global assessments confirm that disease state—not technical noise—dominates variance in both omics layers, validating the dataset for downstream differential and integrative analyses.

### 2.2. Transcriptomic Alterations Indicate an Interferon-Driven, Neutrophil-Skewed Immune Signature

**Figure 2:**
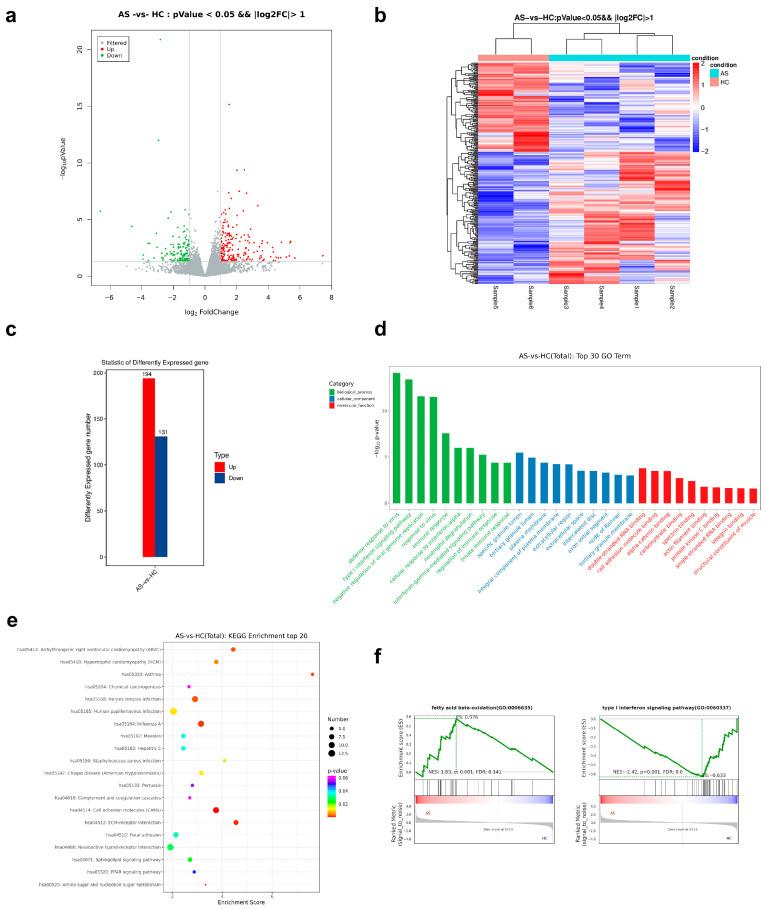
Differential gene expression landscape and pathway enrichment in ankylosing spondylitis (AS) PBMCs. (**a**) Volcano plot of 11,642 genes; red, 194 up-regulated; green, 131 down-regulated. (**b**) Hierarchical clustering of the 325 DEGs separates AS and HC samples. (**c**) Bar summary of up- and down-regulated genes. (**d**) Top-30 GO terms enriched among DEGs (−log_10_ adj-p). (**e**) KEGG functional classification of DEGs. (**f**) GSEA enrichment plots for type I interferon signaling, neutrophil degranulation, and fatty acid β-oxidation.

Using a stringent threshold of |log_2_FC| ≥ 1 and FDR < 0.05, we identified 325 DEGs between AS and HC PBMC (Figure 2a,c). Up-regulated genes were dominated by interferon-stimulated genes (*IFI44L*, *ISG15*, *OAS1*) and neutrophil granule markers (*S100A8*, *S100A9*, *LTF*), while key components of mitochondrial energy metabolism (*ACADM*, *CPT1A*) were down-regulated. Representative differentially expressed genes and their statistics are summarized in Table 1.

Functional enrichment analysis reinforced this immune-metabolic dichotomy. GO terms related to antiviral defense (“defense response to virus”, “type I interferon signaling pathway”, “negative regulation of viral genome replication”) and neutrophil effector functions (“neutrophil degranulation”, “specific granule lumen”, “tertiary granule lumen”) were markedly over-represented (Figure 2d). KEGG analysis showed DEG enrichment in immune system, infectious disease, and signal transduction categories (Figure 2e).

GSEA provided orthogonal validation. Gene sets for type I interferon signaling and neutrophil degranulation were positively enriched, whereas fatty-acid β-oxidation showed significant negative enrichment (Figure 2f). Together, these data depict an AS PBMC transcriptome characterized by heightened interferon tone, activated neutrophils, and suppressed oxidative lipid metabolism. These features that mechanistically align with the metabolomic alterations described above.

### 2.3. Combined GC-/LC-MS Analysis Highlights Bile Acid Dysregulation and Lipid Peroxidation Clusters

**Figure 3:**
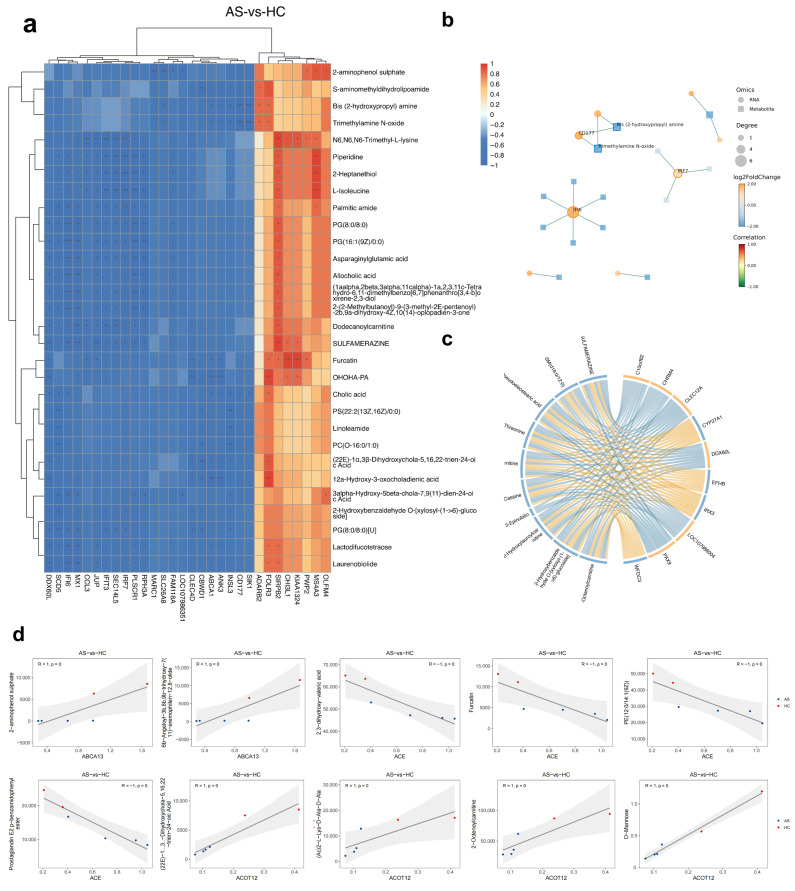
Merged GC-MS and LC-MS (“GCLC”) metabolomic signature differentiates AS from HC. (**a**) Heat map of 110 significant features (25 annotated metabolites); AS clusters separately from HC. (**b**) Spearman network (|ρ| > 0.7) showing a lipid peroxidation module linked inversely to bile acid derivatives. (**c**) Pair-wise correlation matrix of the same metabolites; red = positive, blue = negative. (**d**) KEGG metabolite set enrichment: primary bile acid biosynthesis and fatty acid β-oxidation rank highest. Asterisks denote statistical significance after Benjamini–Hochberg correction: * BH-adjusted *p* < 0.05, ** BH-adjusted *p* < 0.01, *** BH-adjusted *p* < 0.001; ns, not significant.

To maximize chemical coverage, we merged non-redundant features from the GC-MS (volatile/semi-volatile) and LC-MS (polar/lipid) platforms, yielding a unified “GCLC” metabolome. Twenty-five metabolites met the differential threshold (VIP > 1, FC ≥ 1.2, BH-adjusted *p* < 0.05). The heat map (Figure 3a) demonstrates a clear disease-driven signature. Bile acid intermediates such as 3α-hydroxy-5β-chol-7-en-24-oic acid and 12α-hydroxy-5β-chol-6-en-24-oic acid were depleted in AS, whereas lipid peroxidation products (9-oxo-ODE, 12-Z-octadecadienal) and long-chain fatty acid amides (palmitic amide) were elevated. Discriminant metabolites (VIP > 1, FC ≥ 1.2, BH-adjusted *p* < 0.05) are listed in Table 2.

Correlation mapping revealed two antagonistic metabolite modules (Figure 3b,c). A positively co-varying cluster of aldehydes, oxylipins, and fatty acid amides (ρ ≈ 0.75–0.92) showed strong negative correlations (ρ ≈ −0.70) with the bile acid pool, suggesting coordinated regulation or reciprocal depletion between inflammatory lipid mediators and bile acid synthesis.

KEGG pathway enrichment analysis showed that primary bile acid biosynthesis and fatty acid β-oxidation were the most significantly perturbed routes (q < 0.05), followed by linoleic and arachidonic acid metabolism (Figure 3d). These metabolomic findings dovetail with the transcriptomic repression of β-oxidation genes (Figure 2f) and reinforce a model in which chronic inflammation in AS is accompanied by impaired bile acid homeostasis and the accumulation of lipid peroxidation products. Complementary metabolomic platforms supported the LC-MS findings. GC-MS profiling revealed a concordant, although numerically smaller, panel of differential metabolites and pathways (Supplementary Figure S3a–c). An independent LC-MS positive-ion dataset reproduced these alterations and further highlighted glycerophospholipid metabolism as a recurrently perturbed module (Supplementary Figure S4a–c).

### 2.4. Multi-Omics Correlations Reveal Lipid Peroxidation and Bile Acid Hubs That Track Immune Activation

Across the six PBMC profiles, we computed pair-wise Spearman correlations between the 325 DEGs and the 110 differential metabolites. After Benjamini–Hochberg correction, 112 gene–metabolite pairs surpassed the significance cut-off (|ρ| > 0.65, q < 0.05)

**Figure 4:**
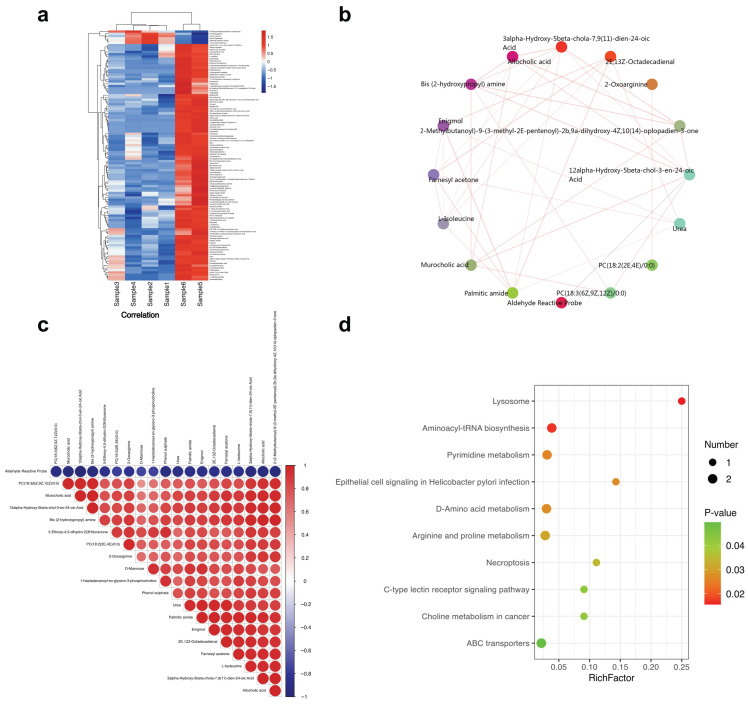
Integrated transcriptome-metabolome analysis pinpoints a coordinated lipid immune axis in AS (**a**) Spearman heat map of 30 DEGs vs. 110 features (25 annotated metabolites) (|ρ| > 0.65, q < 0.05). (**b**) Correlation network filtered at |ρ| > 0.75; circles = metabolites, squares = genes. (**c**) Chord diagram showing oxylipin/aldehyde (orange) and bile acid (blue) modules. (**d**) Representative scatter plots illustrating positive (*ABCA13*-3-hydroxypropyl-amine, ρ = 0.92) and negative (*ACE*-palmitic amide, ρ = −0.88; *ACOT12*-cholic acid, ρ = −0.83) gene–metabolite relationships; shaded areas show 95% confidence intervals.

The resulting heat map (Figure 4a) separated into two antagonistic blocks. The first block contained interferon-stimulated and neutrophil granule genes (*IFI44L*, *ISG15*, *S100A8/9*, *LTF*) that correlated positively with a suite of oxidized lipids and fatty acid amides (e.g., 12-Z-octadecadienal, palmitic amide) but negatively with primary bile acids. The second block featured lipid catabolism genes (*ACOT12*, *CPT1A*, *ABCA13*) displaying the inverse pattern with positive links to cholic and chenodeoxycholic derivatives and negative links to the pro-inflammatory oxylipins.

Network analysis (Figure 4b) highlighted palmitic amide and 12-Z-octadecadienal as hub metabolites (degree ≥ 6), while *ABCA13*, *ACE*, and *ACOT12* emerged as the top gene hubs. The chord diagram (Figure 4c) visualizes these dual modules, underscoring a reciprocal relationship between bile acid homeostasis and lipid peroxidation pathways.

Selected scatter plots (Figure 4d) confirmed the tight correlations: *ABCA13* scaled positively with 3-hydroxypropyl-amine (ρ = 0.92, q = 0.004), whereas *ACE* inversely tracked palmitic amide (ρ = −0.88, q = 0.009). These associations suggest that vascular signaling genes interface with inflammatory lipid mediators and that impaired *ACOT12* parallels bile acid depletion.

**Figure 5:**
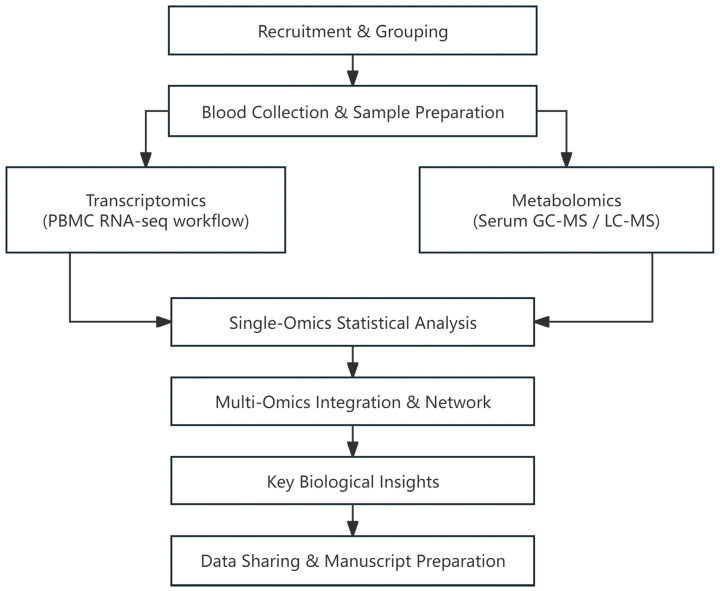
Overview of the experimental and analytical workflow for the sibling-paired dual-omics study.

Taken together, the integrative analysis indicates that chronic interferon/neutrophil activation in AS is metabolically coupled to accumulated lipid peroxidation products and attenuated bile acid biosynthesis, providing a mechanistic bridge between the transcriptomic and metabolomic layers. The overall workflow is summarized in Figure 5 and can be readily applied to larger validation cohorts.

## 3. Discussion

In this pilot investigation, we integrated PBMC RNA-seq with untargeted GC-MS and LC-MS profiling to delineate an immune metabolic signature that cleanly separates AS patients from age-matched HCs. Although the cohort is small, the concordant stratification across multiple omics layers (Figure 1) and the coherent gene–metabolite correlations (Figure 4 and Figure 5) argue that the observed signals are disease driven rather than technical artefacts. Below, we contextualize each major finding and propose a mechanistic model linking chronic inflammation to lipid and bile acid dysregulation in AS.

### 3.1. A Systemic Type I Interferon/Neutrophil Program Dominates the AS PBMC Transcriptome

The RNA-seq data revealed a robust up-regulation of interferon-stimulated genes (ISGs) and neutrophil granule markers (Figure 2a–d). While *IL-17*/*IL-23* signaling is classically emphasized in spondylarthritis, accumulating evidence shows that type I interferons and neutrophil extracellular traps fuel enthesitis and bone remodeling [14,15]. Elevated *IFI44L*, *ISG15*, and *OAS1* mirror transcriptomic patterns reported in psoriatic arthritis and severe COVID-19, suggesting convergent antiviral-like activation [16,17]. Simultaneously, strong induction of *S100A8*/*S100A9* and LTF underscores the contribution of primed neutrophils, which have been linked to radiographic progression in AS. The GSEA enrichment of “type-I interferon signaling pathway” and “neutrophil degranulation” (Figure 2f) therefore positions antiviral defense and granulocyte activation as central, interconnected drivers of systemic inflammation in our subjects.

### 3.2. Suppression of Fatty Acid β-Oxidation and Accumulation of Lipid Peroxidation Products

Down-regulation of mitochondrial lipid catabolism genes (*ACADM*, *CPT1A*, *ACOT12*) coincided with negative GSEA enrichment of “fatty acid β-oxidation” and elevated circulating aldehydes, oxylipins, and long-chain fatty acid amides (Figure 2f and Figure 4a). These metabolites are hallmarks of lipid peroxidation and oxidative stress; they modulate neutrophil chemotaxis and can amplify NF-κB signaling. The bidirectional correlations depicted in Figure 4a–c reinforce this link. Oxidized lipids correlated positively with neutrophil/interferon genes and negatively with β-oxidation transcripts, whereas primary bile acids showed the opposing pattern [18]. Collectively, the data suggest that inefficient mitochondrial β-oxidation might favor peroxisomal or non-enzymatic oxidation, thereby expanding an inflammatory lipid pool that feeds back into innate immune activation.

### 3.3. Bile Acid Depletion as a Putative Metabolic Checkpoint in AS

Primary bile-acid species (e.g., cholic acid, 12α-hydroxy-5β-chol-6-en-24-oic acid) were uniformly reduced in AS PBMCs and inversely correlated with neutrophil granule transcripts (Figure 3a–c and Figure 4a–c). Beyond fat absorption, bile acids act as immuno-modulators through *FXR* and *TGR5* receptors, suppressing *NLRP3* inflammasome activity and *IL-17* production [19]. Reduced bile- acid availability could therefore remove a brake on pathologic innate immunity. Whether this depletion reflects hepatic synthesis defects, microbial dysbiosis, or altered enterohepatic circulation in AS remains to be established, but it offers a mechanistic bridge between gut involvement and systemic inflammation that characterizes the spondylarthritis spectrum.

### 3.4. Gene–Metabolite Hubs Suggest Novel Biomarkers and Therapeutic Avenues

The integrative network pinpointed palmitic amide, 12-Z-octadecadienal and *ABCA13*/*ACE*/*ACOT12* as correlation hubs (Figure 4b–d). Palmitic amide is an endogenous PPAR-α agonist with anti-nociceptive properties [20]; its elevation may represent a compensatory response to inflammation and pain. Conversely, 12-Z-octadecadienal, a reactive α-oxoaldehyde, can form protein adducts and drive oxidative stress. The positive relationship between *ABCA13* (a lipid transporter linked to macrophage lipid handling) and 3-hydroxypropyl-amine suggests altered cholesterol/phospholipid export in AS PBMC immune cells. Such multi-layer hubs merit evaluation as composite biomarkers for disease activity and as potential targets for host-directed therapy (e.g., aldehyde scavengers, *FXR* agonists, *PPAR* modulators).

### 3.5. Limitations and Future Directions

Our study is limited by the small, male-only cohort and cross-sectional design, which preclude causal inference and sex-specific analyses. PBMC omics provide systemic snapshots but cannot resolve tissue resident processes in the spine or gut. Future work should (i) validate the lipid peroxidation–bile acid axis in larger, longitudinal cohorts; (ii) examine how *TNF-α* or *IL-17* blockade modulates the identified gene–metabolite modules; (iii) integrate microbiome and single-cell data to assign cellular and microbial sources to the circulating signatures; and (iv) perform functional assays to test whether restoring bile acid signaling or enhancing mitochondrial β-oxidation attenuates interferon-mediated neutrophil activation.

## 4. Conclusions

In this sibling-paired PBMC study, we combined strand-specific RNA-seq with untargeted GC-MS and LC-MS to capture ankylosing spondylitis-specific molecular changes. A total of 325 differentially expressed genes were detected, with interferon-stimulated and neutrophil granule transcripts (e.g., *IFI44L*, *ISG15*, *S100A9*) up-regulated and key β-oxidation enzymes (*ACADM*, *CPT1A*, *ACOT12*) down-regulated. Metabolomic integration yielded 110 non-redundant discriminatory metabolites whose abundances alone separated all four AS patients from their healthy brothers. Long-chain oxylipins and fatty-acid amides accumulated, whereas primary bile acid intermediates were depleted. Cross-layer correlation condensed these findings into two antagonistic hubs: pro-oxidative lipids tightly aligned with the interferon–neutrophil gene cluster and bile acid species inversely linked to lipid catabolism genes. Together, these results define an interferon-linked lipid–bile acid axis that mechanistically joins immune activation to metabolic stress in AS and nominates a compact panel of transcripts and metabolites—palmitic amide, 12-Z-octadecadienal, cholic-acid derivatives, *IFI44L*, *S100A9*, *ACADM*—for prospective validation as composite biomarkers and therapeutic targets. Expansion to larger, longitudinal cohorts and cell-resolved assays will be the next step toward clinical translation.

## 5. Materials and Methods

### 5.1. Study Design and Subjects

This exploratory sibling-paired dual-omics study enrolled three biological brother pairs (AS = 4, healthy = 2) from two tertiary rheumatology clinics between May 2023 and October 2024. Two pairs carried a confirmed diagnosis of AS according to the 2009 ASAS classification criteria, whereas the third pair were *HLA-B27*-positive but clinically healthy. In each AS pair, one brother exhibited advanced structural damage (modified Stoke Ankylosing Spondylitis Spinal Score, mSASSS > 40; history of spinal corrective surgery) and was designated as severe. The other had mild radiographic changes (mSASSS < 10) and was designated mild. None of the six volunteers had received biologics, systemic glucocorticoids, statins, antibiotics, probiotics, or disease-modifying antirheumatic drugs within 8 weeks before sampling. Exclusion criteria included (i) inflammatory bowel disease or psoriasis; (ii) acute infection within 4 weeks; (iii) body mass index (BMI) > 30 kg m^−2^; (iv) serum creatinine > 120 µmol L^−1^ or ALT/AST > 2 × Upper Limit of Normal (ULN); and (v) active smoking (>5 cigarettes day^−1^) or alcohol abuse. An overview of the sampling, acquisition, and integration workflow is provided in Figure 5.

### 5.2. Clinical Assessment, Data Collection, and Ethics

Demographic and clinical variables, including sex, age, surgical history, *HLA-B27* status, mSASSS, and Bath Ankylosing Spondylitis Disease Activity Index (BASDAI), were recorded on the day of blood collection. Detailed participant characteristics are summarized in Table 3.

The study adhered to the Declaration of Helsinki and was approved by the Ethics Committee of Chinese PLA General Hospital (approval No. ChiCTR2400090375). Written informed consent was obtained from all participants prior to enrolment.

### 5.3. RNA Extraction, Library Construction, and Sequencing

Total RNA was extracted using TRIzol reagent (Invitrogen, Carlsbad, CA, USA) according to the manufacturer’s protocol. RNA purity and concentration were assessed on a NanoDrop 2000 spectrophotometer (Thermo Scientific, Waltham, MA, USA), and integrity was verified on an Agilent 2100 Bioanalyzer (Agilent Technologies, Santa Clara, CA, USA). Samples meeting purity (A_260_/_280_ ≈ 2.0), quantity, and integrity (RIN ≥ 8.0) criteria proceeded to library preparation. Ribosomal RNA was depleted with the Ribo-off rRNA Depletion Kit (Vazyme, Nanjing, China), and sequencing libraries were constructed using the VAHTS Universal V6 RNA-seq Library Prep Kit (Vazyme) following the manufacturer’s instructions. Libraries were quantified using Qubit dsDNA HS and qPCR and then pooled equimolarly. Transcriptome sequencing was performed by OE Biotech Co., Ltd. (Shanghai, China) on an Illumina NovaSeq 6000 platform (PE150).

### 5.4. RNA Sequencing Analysis Process

Raw 150 bp paired-end reads (approximately 45 million per sample) were first quality filtered and adapter trimmed using fastp, yielding about 42 million high-quality clean reads per sample. Clean reads were aligned to the GRCh38 reference genome with HISAT2. Gene-level counts were generated using HTSeq-count, and expression was normalized to fragments per kilobase of transcript per million mapped reads (FPKM). Principal component analysis (PCA) to evaluate sample clustering and biological reproducibility was conducted in R (v3.2.0).

### 5.5. Differential Gene Expression Analysis

Differential expression between AS and HC groups was assessed with DESeq2, applying thresholds of FDR < 0.05 and |log_2_FC| ≥ 1 to define DEGs to define significantly differentially expressed genes (DEGs). Hierarchical clustering of DEGs was performed in R (v3.2.0) to visualize group-specific expression patterns. A radar plot of the top 30 up- and down-regulated DEGs was generated using the ggradar package. Functional enrichment analyses, including Gene Ontology, KEGG, Reactome, and WikiPathways, were conducted using hypergeometric testing; significant terms were displayed as bar charts, chord diagrams, and bubble plots in R. Gene set enrichment analysis (GSEA) was performed using GSEA Desktop v4.3.2 (Broad Institute) with gene sets from MSigDB v7.5.1, ranking genes by log_2_ fold change to detect enrichment of predefined gene sets.

### 5.6. Serum Metabolite Extraction and GC-MS Acquisition

Serum (50 µL) was thawed on ice and spiked with L-2-chlorophenylalanine (0.3 mg/mL in methanol, 10 µL) as an internal standard. Proteins were precipitated with 200 µL ice-cold methanol/acetonitrile (2:1, *v*/*v*), vortexed (1 min), sonicated in an ice-water bath (10 min), and kept at −20 °C for 30 min. After centrifugation (13,000 rpm, 10 min, 4 °C), the supernatant was transferred and dried under vacuum. Derivatization proceeded with methoxyamine hydrochloride (15 mg/mL in pyridine, 30 µL, 37 °C, 90 min) followed by silylation with BSTFA + 1% TMCS (30 µL) and n-hexane (30 µL) at 70 °C for 60 min; samples were equilibrated 30 min at room temperature prior to analysis.

GC–MS was performed on an Agilent 7890B GC coupled to a 5977B MSD using a DB-5MS capillary column (30 m × 0.25 mm × 0.25 µm) with the following parameters: injection, 1 µL, splitless, inlet 260 °C; carrier gas, helium (1.0 mL/min); oven, 60 °C (0.5 min), 8 °C/min to 125 °C, 4 °C/min to 210 °C, 5 °C/min to 270 °C, 10 °C/min to 305 °C (hold 3 min); EI 70 eV; ion source 230 °C; quadrupole 150 °C; full-scan *m*/*z* 50–500; solvent delay 5 min.

A pooled QC was injected every 10 samples and solvent blanks between batches. Raw files were processed in MS-DIAL (peak picking, deconvolution, alignment, gap filling); library matching used NIST/Fiehn libraries with retention-index anchoring. Features with QC-RSD > 30% were removed prior to statistics.

### 5.7. Serum Metabolite Extraction and LC-MS Acquisition

Frozen serum aliquots (100 µL) were thawed on ice, vortexed, and subject to protein precipitation with 400 µL chilled methanol:acetonitrile (1:1, *v*/*v*) containing 5 µM sulfachloropyridazine as internal standard. After vortexing (30 s), samples were incubated at −20 °C for 1 h and centrifuged (16,000× *g*, 15 min, 4 °C). Supernatants (350 µL) were vacuum dried and reconstituted in 100 µL 50% acetonitrile. A pooled quality control (QC) sample (aliquot of each extract) was injected every 5 runs. Instrument drift was corrected using QC-RLSC, and lock-mass calibration (*m*/*z* = 131.040) was applied at 20-scan intervals.

Chromatography: Vanquish UHPLC (Thermo Fisher) with a Waters HSS T3 column (2.1 × 100 mm, 1.8 µm, 40 °C). Mobile phase A: 0.1% formic acid-water; B: 0.1% formic acid-acetonitrile. Gradient: 0–1 min 99% A; 1–9 min linear to 1% A; 9–12 min 1% A; 12.1–15 min re-equilibrate 99% A. Flow 0.3 mL min^−1^; injection 3 µL.

Detection: Q Exactive Plus Orbitrap (Thermo Fisher) with HESI-II source. Positive/negative modes acquired separately: sheath gas 35, aux gas 10, spray voltage ± 3.5 kV, capillary 320 °C. Full MS scan (*m*/*z* 70–1050, 70,000 FWHM) followed by data-dependent MS^2^ (17,500 FWHM, stepped NCE 20/40/60).

Raw files were centroided and converted to mzML (MSConvert). A blank, pooled QC and Pierce LTQ ESI positive calibration mix were used to monitor carry-over and mass accuracy.

### 5.8. Metabolomics Data Processing and Annotation

XCMS v3.20 (centWave algorithm) performed peak picking (ppm 10, peak width 10–60 s), retention time correction (obiwarp), and grouping (bw 5 s). CAMERA v1.52 assigned isotopes/adducts. Intensities were log_2_ transformed, Pareto scaled, and filtered for features present in ≥4 samples. QC-pooled features with RSD > 30% were removed, yielding 9655 ions. Compound identities were assigned by matching experimental *m*/*z* and MS^2^ spectra to HMDB, mzCloud, and LipidBlast (mass tolerance 10 ppm; dot-product ≥ 0.6). Confidence levels followed the Metabolomics Standards Initiative (MSI).

Differential metabolites were computed in R using limma v3.58.1 with blocking factor PairID. VIP scores from orthogonal partial least squares discriminant analysis (OPLS-DA) (ropls v1.32) complemented univariate *p*-values. Significance: VIP > 1, FC ≥ 1.2, BH-adjusted *p* < 0.05.

### 5.9. Gene Set Enrichment Analysis

DEG lists were input to clusterProfiler v4.10 (org.Hs.eg.db v3.17). Over-representation analysis used GO BP, KEGG, and Reactome (q < 0.05, background = all detected genes). Metabolic features were annotated to KEGG compound IDs; mummichog pathway analysis (MetaboAnalyst v5.0) used 10,000 permutations. Shared pathways were visualized with GOplot (circos) and ggVennDiagram.

### 5.10. Differential Feature Selection and Data Normalization

After separate single-omics analyses, candidate variables were first filtered to reduce noise prior to integration. Genes that met the criteria |log_2_ fold-change| ≥ 1 together with a Benjamini–Hochberg-adjusted false discovery rate (FDR) < 0.05 (Section 5.5) were retained as differentially expressed genes (DEGs). Metabolites were deemed differential when VIP > 1, FC ≥ 1.2, and BH-adjusted *p* < 0.05 (Section 5.8). The resulting gene counts were variance-stabilizing transformed with DESeq2 (v1.38.3) and centered to a zero mean for each gene, whereas metabolite peak intensities were log_10_-transformed followed by Pareto scaling with MetaboAnalystR (v3.2). These steps harmonized distributional properties across the two data layers and ensured that subsequent statistics were not dominated by scale differences.

### 5.11. Combined Transcriptome–Metabolome Analysis

Spearman correlation coefficients (r) were calculated between each differentially expressed gene (DEG; |log_2_FC| ≥ 1, FDR < 0.05) and each differentially abundant metabolite (DEM; VIP > 1, FC ≥ 1.2, BH-adjusted *p* < 0.05) across the six matched samples using base R. Associations with |r| ≥ 0.70 and Benjamini–Hochberg FDR < 0.05 were deemed significant. A correlation heatmap and full correlation matrix of DEGs versus DEMs were plotted with pheatmap (v1.0.12). Significant gene–metabolite pairs were exported to Cytoscape (v3.10.0) to construct an association network, with nodes colored by molecule type (gene or metabolite) and sized by degree.

### 5.12. Pathway Enrichment and KEGG Mapping

DEGs and DEMs were each subjected to pathway enrichment analysis, and their shared pathways were identified. Enriched pathways were mapped to Kyoto Encyclopedia of Genes and Genomes (KEGG) using a custom R script developed by OE Biotech Co., Ltd. The resulting KEGG pathway maps highlight molecular circuits concurrently perturbed at both the transcript and metabolite levels.

### 5.13. Statistical Considerations

To account for the sibling design, all between-group tests incorporated a family blocking factor (PairID). Multiple testing was controlled using the Benjamini–Hochberg FDR. For supervised metabolomics models (OPLS-DA), we report R^2^Y and Q^2^ with label-permutation tests (*n* = 2000) to assess over-fitting; leave-one-out cross-validation (LOOCV) was additionally used for correlation stability. Where applicable, we provide effect sizes and 95% confidence intervals alongside p/FDR values.

## Figures and Tables

**Table 1:** Top ten up-regulated and ten down-regulated DEGs in AS versus HC (|log_2_FC| ≥ 1, FDR < 0.05).

Gene Name	Log2FC	Fold Change	Adjusted *p*-Value (BH-FDR)	Regulation
*SIK1*	3.34	10.16	5.86 × 10^−7^	Up
*TMEM191B*	2.89	7.40	7.00 × 10^−5^	Up
*LOC107986351*	2.63	6.19	4.59 × 10^−8^	Up
*CD177*	2.49	5.62	3.95 × 10^−10^	Up
*IFI6*	2.42	5.35	1.71 × 10^−6^	Up
*RPH3A*	2.16	4.48	3.08 × 10^−8^	Up
*MX1*	2.03	4.08	4.42 × 10^−6^	Up
*SCD5*	2.01	4.04	4.37 × 10^−10^	Up
*IFIT3*	2.01	4.02	2.94 × 10^−5^	Up
*OSM*	1.76	3.38	4.89 × 10^−5^	Up
*MS4A3*	−1.33	0.40	3.65 × 10^−5^	Down
*PTPRF*	−1.37	0.39	1.24 × 10^−4^	Down
*CAMP*	−1.73	0.30	5.13 × 10^−5^	Down
*CHI3L1*	−2.16	0.22	2.16 × 10^−6^	Down
*OLFM4*	−2.33	0.20	7.52 × 10^−6^	Down
*FOLR3*	−2.82	0.14	1.28 × 10^−21^	Down
*PWP2*	−2.95	0.13	9.85 × 10^−13^	Down
*MYO18B*	−3.08	0.12	1.61 × 10^−4^	Down
*TBC1D3*	−4.64	0.04	4.05 × 10^−5^	Down
*ADARB2*	−6.62	0.01	1.87 × 10^−6^	Down

**Table 2:** Top six up-regulated and ten down-regulated metabolites in the AS versus HC comparison identified using LC-MS, plus three additional significantly altered metabolites detected with GC-MS.

Data Class	Metabolites	VIP	Fold Change	Adjusted *p*-Value (BH-FDR)	Regulation
GC	D-Mannose	8.49	0.26	3.38 × 10^−2^	Down
GC	Urea	6.57	0.77	2.34 × 10^−2^	Down
GC	L-Alpha-aminobutyric acid	1.03	0.47	4.07 × 10^−2^	Down
LC	Leucyl-leucine	1.14	3.27	3.96 × 10^−2^	Up
LC	Hexyl heptanoate	1.58	3.18	1.99 × 10^−2^	Up
LC	Aldehyde reactive probe	3.69	3.08	1.31 × 10^−2^	Up
LC	Tri-N-acetylchitotriose	2.19	2.97	1.46 × 10^−2^	Up
LC	3,4-Dehydrothiomorpholine-3-carboxylate	1.57	2.24	2.76 × 10^−2^	Up
LC	C16 Sphinganine	2.92	1.33	4.69 × 10^−2^	Up
LC	2-(2-methylbutanoyl)-9-(3-methyl-2E-pentenoyl)-2b,9a-dihydroxy-4Z,10(14)-oplopadien-3-one	3.22	0.02	2.59 × 10^−4^	Down
LC	6b-Angeloyl-3b,8b,9b-trihydroxy-7(11)-eremophilen-12,8-olide	1.14	0.02	4.37 × 10^−3^	Down
LC	PG(8:0/8:0)	2.22	0.02	2.51 × 10^−4^	Down
LC	(1aalpha,2beta,3alpha,11calpha)-1a,2,3,11c-Tetrahydro-6,11-dimethylbenzo [6,7]phenanthro [3,4-b]oxirene-2,3-diol	1.64	0.01	1.09 × 10^−4^	Down
LC	2-Aminophenol sulphate	1.06	0.01	4.19 × 10^−4^	Down
LC	PG(8:0/8:0) [U]	1.07	0.00	2.87 × 10^−5^	Down
LC	Lactodifucotetraose	1.22	0.00	1.54 × 10^−4^	Down
LC	Niveusin C	2.67	0.00	9.92 × 10^−3^	Down
LC	SM(d18:0/12:0)	1.47	0.00	4.73 × 10^−3^	Down
LC	3-Epinobilin	1.53	0.00	1.71 × 10^−2^	Down

**Table 3:** Participant demographics and clinical data.

Number	Group	F/M	Age	Surgery History	HLA-B27	mSASSS	BASDAI
Sample 1	AS	M	49	YES	+	32	4.5
Sample 2	AS	M	51	NO	+	10	2.3
Sample 3	AS	M	40	YES	+	45	5.1
Sample 4	AS	M	43	NO	+	12	2.6
Sample 5	HC	M	41	NO	−	−	−
Sample 6	HC	M	41	NO	−	−	−

## Data Availability

RNA-seq FASTQ files will be deposited to the SRA (BioProject ID to be provided upon acceptance). LC-/GC-MS raw files and processed tables will be deposited to MetaboLights/Metabolomics Workbench and made public upon acceptance. Temporary reviewer access can be provided upon request.

## Supplementary Materials

The following supporting information can be downloaded at: https://www.mdpi.com/article/10.3390/ijms26167919/s1.

## Abbreviations

AS	Ankylosing Spondylitis
HC	Healthy Controls
PBMCs	Peripheral Blood Mononuclear Cells
GC-MS	Gas Chromatography–Mass Spectrometry
LC-MS	Liquid Chromatography–Mass Spectrometry
mSASSS	Modified Stoke Ankylosing Spondylitis Spinal Score
ULN	Upper Limit of Normal
BMI	Body Mass Index
BASDAI	Bath Ankylosing Spondylitis Disease Activity Index
PCA	Principal Component Analysis
OPLS-DA	Orthogonal Partial Least Squares Discriminant Analysis
GSEA	Gene Set Enrichment Analysis
QC	Quality Control
MSI	Metabolomics Standards Initiative
DEM	Differentially Abundant Metabolite
DEGs	Differentially Expressed Genes
KEGG	Kyoto Encyclopedia of Genes and Genomes
