# Supplementary material for: Interferon-Linked Lipid and Bile Acid Imbalance Uncovered in Ankylosing Spondylitis in a Sibling-Controlled Multi-Omics Study
Source: Int J Mol Sci. 2025 Aug 16;26(16):7919. doi: 10.3390/ijms26167919 (PMC12386361; doi:10.3390/ijms26167919)
Supplement: Supplementary file 1 [file ijms-26-07919-s001.zip › ijms-3768830-supplementary.pdf]

a

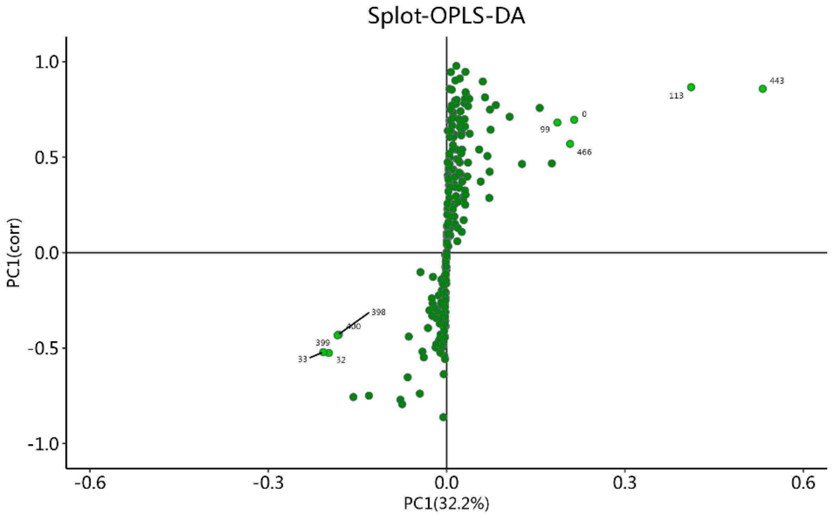

Supplementary Figure S1. GC-MS S-plot pinpointing ions with the highest |p| and |p(corr)|.

a

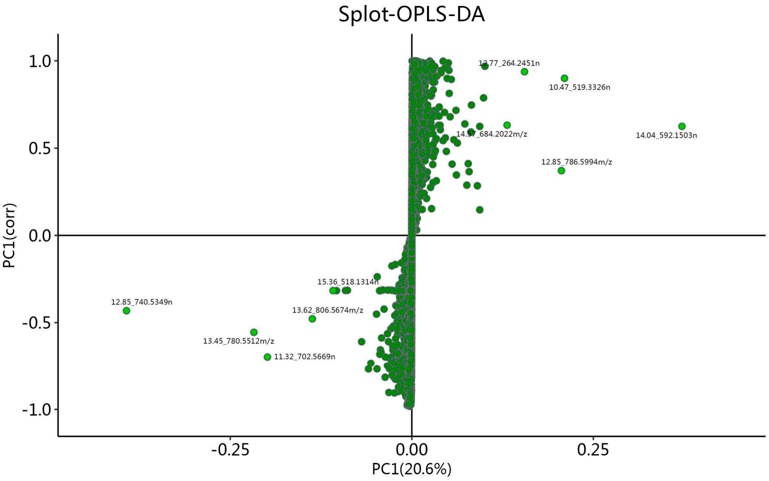

Supplementary Figure S2. LC-MS S-plot pinpointing ions with the highest |p| and |p(corr)|.

a

b

c

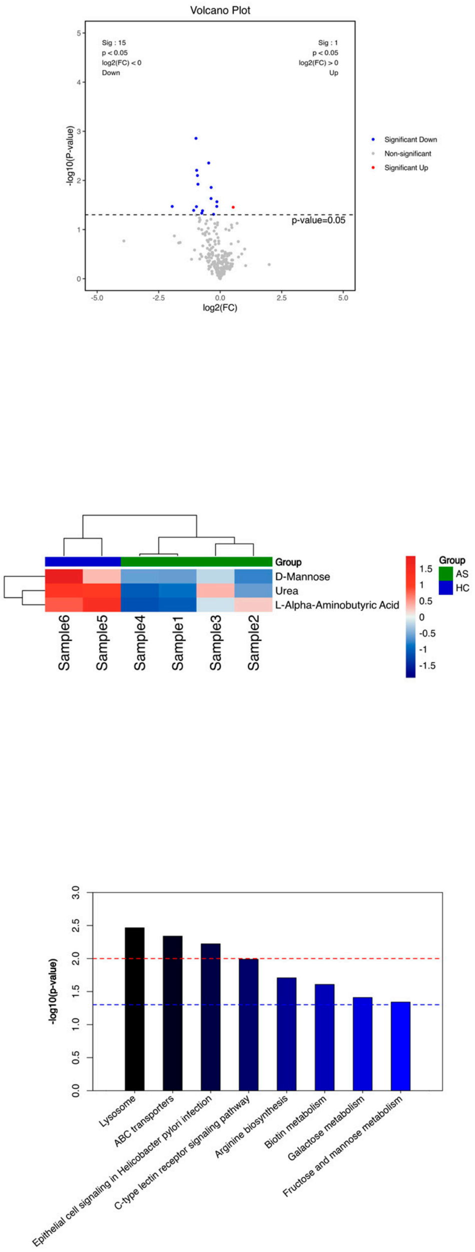

Supplementary Figure S3.GC-MS metabolomic signatures distinguishing AS patients from HCs. (**a**) Volcano plot of 116 detected metabolites. Blue points mark 18down-regulated metabolites (|FC≥1.2, *p*< 0.05); no metabolites were up-regulated. Horizontal and vertical dashed lines denote *p*= 0.05 and FC =1.2, respectively. (**b**) Hierarchical clustering heat map of the 25 most significant metabolites (Euclidean distance, Ward linkage). Sample-level side bars indicate clinical group (red = AS, green = HC). (**c**) Over-representation analysis of the differential metabolite set (MetaboAnalyst, KEGG pathways). Bars show -$\log_{10}(p)$; the red dotted line marks *p*= 0.05. Black bars represent pathways enriched in all significant metabolites, whereas blue bars correspond to partially enriched pathways.

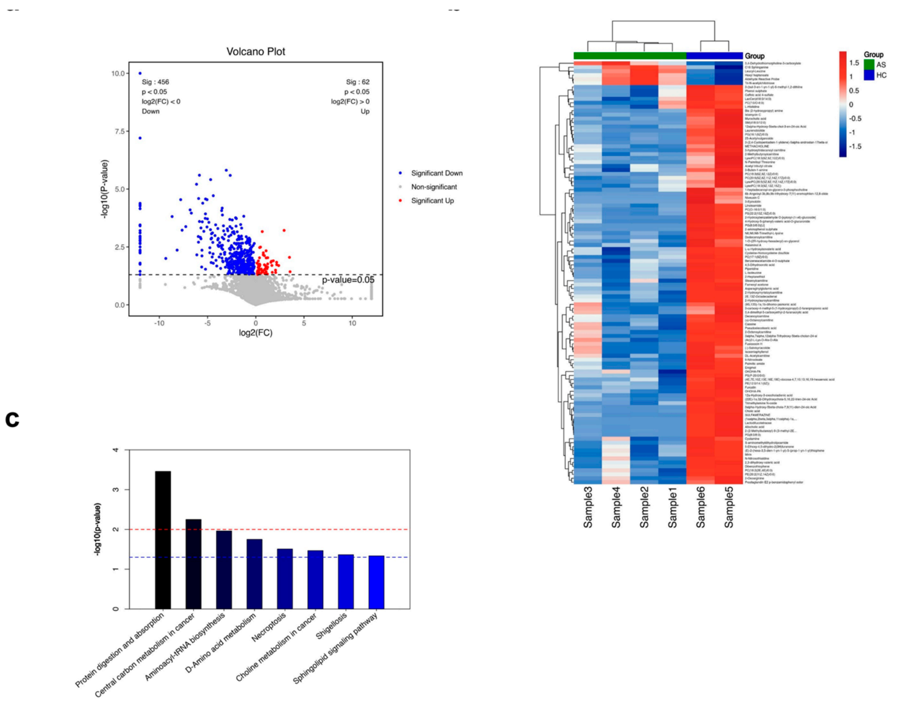

Supplementary Figure S4. LC-MS metabolomic signatures distinguishing patients with AS from HCs. (**a**) Volcano plot of 402 LC-MS features. Blue points indicate 82 down-regulated metabolites, and red points indicate 17 up-regulated metabolites (criteria as in S1A).(**b**) Heat map of the top 50 differential LC-MS features, organized as in S1B. (**c**) KEGG pathway enrichment of the LC-MS differential set. Plot elements and color scheme are identical to S1C.
